# Visible–Infrared Dual-Modal Monitoring System for Overlap Defects in Wire Arc Additive Manufacturing

**DOI:** 10.3390/ma19050899

**Published:** 2026-02-27

**Authors:** Weixin Wang, Peng Gao, Dongli Chen, Runzhen Yu, Hongwei Kang, Zhuang Zhao

**Affiliations:** 1 School of Mechanical Science and Engineering, Huazhong University of Science and Technology, Wuhan 430074, China; wxwang2014@126.com; 2 Hubei Hongyang Sanjiang Aerospace Electromechanical Co., Ltd., Xiaogan 432100, China; yuro94@126.com (R.Y.); kanghw0824@163.com (H.K.); 3 State Key Laboratory of Extreme Environment Optoelectronic Dynamic Testing Technology and Instrument, Nanjing University of Science and Technology, Nanjing 210094, China; chendongli0323@163.com; 4 Jiangsu Key Laboratory of Visual Sensing & Intelligent Perception, Nanjing University of Science and Technology, Nanjing 210094, China

**Keywords:** visible–infrared image, wire arc additive manufacturing, overlap defect, deep learning

## Abstract

This paper proposes a dual-modal monitoring system combining visible and infrared imaging to enhance overlap defect detection in wire arc additive manufacturing (WAAM) based on cold metal transfer (CMT) welding for multi-pass builds. Traditional single-modal approaches, primarily relying on melt pool imagery, are often hindered by arc light and spatter interference, which can compromise detection accuracy. In this work, overlap defect refers to insufficient overlap between adjacent tracks, and the dataset is created by inducing overlap defects through inter-track spacing in multi-pass deposition. The proposed dual-modal strategy mitigates these challenges and significantly improves detection precision. A dual-input convolutional neural network model named Multimodal Mutual Fusion Network (MMFNet) was designed, fusing visible and infrared data at the feature level to achieve a prediction accuracy of 98.34%. Comparative experiments with single-modal models demonstrate the superiority of the proposed approach, with single-modal accuracies of only 95.76% (infrared) and 92.85% (visible light). The proposed system provides a robust solution for monitoring of overlap defects in WAAM in the studied multi-pass setting, highlighting the potential of dual-modal systems for improving quality control in additive manufacturing processes.

## 1. Introduction

Wire arc additive manufacturing (WAAM), a subset of additive manufacturing (AM), has emerged as a transformative technology for fabricating complex geometric shapes and customized parts. WAAM utilizes welding processes, such as cold metal transfer (CMT), to deposit material layer by layer, enabling the production of large-scale components with high deposition rates. However, ensuring quality in WAAM poses significant challenges, particularly in detecting defects during the deposition process. Traditionally, the industry relies on post-weld inspections, which have several disadvantages [1]. Detected defects typically result in either reworking or scrapping the component, leading to considerable production waste [2]. Moreover, this method is prone to oversights that can cause failures or safety hazards in subsequent uses of the components [3]. Therefore, real-time monitoring, especially of the melt pool, is essential to mitigate these risks [4].

Online quality monitoring in WAAM generally follows two approaches: analyzing the relationship between welding parameters (e.g., current and voltage) and quality targets (e.g., defect presence) [5,6], and examining the correlation between in situ data from various modalities and these quality targets. In situ data, acquired via non-contact sensors such as infrared cameras for thermal fields [7,8,9], audio cards for arc sounds [10,11,12], and three-dimensional scanning equipment for morphology [13], provides a stronger statistical correlation than welding parameters alone. This data more accurately reflects the physical and chemical changes during the deposition process, thereby enhancing model learning and improving prediction accuracy.

Furthermore, the acquisition and processing of in situ data enable timely feedback to the WAAM control system, facilitating immediate adjustments and optimizations within the process. While mainstream online monitoring in intelligent manufacturing still predominantly uses single-mode imaging, such as visible cameras to observe melt pool dynamics [14,15,16,17,18,19,20], these systems, effective under controlled conditions, often falter in AM processes like CMT due to severe interference from arc light and spatter.

The aforementioned studies underscore significant limitations in using single-sensor systems for online quality monitoring in WAAM. These systems either fail to achieve real-time monitoring or are affected by noise interference, resulting in low prediction accuracy and poor robustness of single-mode monitoring models. These limitations underscore the urgent need for more robust and effective monitoring methods. In recent years, multimodal learning has become a prominent area of interest within the field of deep learning [21,22]. This approach leverages data from diverse modalities, significantly enhancing a model’s capacity to comprehend and process tasks by integrating varied informational inputs. Its applications span several domains, including autonomous driving and virtual reality [23,24,25], proving its versatility and efficacy. Similarly, multimodal learning can be strategically applied to online quality monitoring in WAAM to overcome the inherent constraints associated with single-modality data. Numerous scholars have explored the integration of multimodal information within the welding domain [26], utilizing combinations such as image and sound [27,28,29,30], image and spectroscopy [31,32,33], and spectroscopy with photodiode data to discern welding characteristics [34,35]. However, incorporating non-visual information into CNN often requires preprocessing, and in some cases, manual feature extraction, complicating their application in real-time monitoring. Additionally, when selecting different modalities as source data, the alignment of information requires meticulous design. This includes synchronizing and aligning data from various sensors and appropriately truncating sequence lengths to manage one-dimensional sound sequences, two-dimensional images, and three-dimensional morphological features. Despite these efforts, the combination of data across different dimensions remains abstract and challenging to interpret specifically within the context of WAAM.

The latest advancements in imaging technology have facilitated the integration of multiple imaging modes, thereby enhancing detection capabilities under challenging conditions. Unlike using different sensors, the combination of multiple imaging technologies can easily ensure synchronous data acquisition, capable of characterizing the same melt pool state. Also, as 2D image data, they can be directly fed into the CNN without preprocessing. For example, the combination of infrared and visible light imaging provides a complementary perspective, widely used in large-scale scenarios such as autonomous driving [36]. Despite the considerable potential of multimodal imaging systems, their application in WAAM still faces significant gaps [37,38], particularly in the study of specific defects such as overlap defects in the narrow field of view. In this paper, an overlap defect refers to insufficient overlap between adjacent tracks, which results in incomplete inter-track bonding. The interactions between material layers make it crucial to detect these defects. The combination of visible and infrared images can capture thermal dynamics obscured by interference from the visible spectrum.

This study investigates a dual-modal approach that combines visible and infrared imaging for melt pool defect detection in WAAM, aiming to improve in-process defect monitoring by leveraging complementary spectral information. The integration of these two modalities aims to overcome the traditional limitations posed by single-mode systems, allowing for a more comprehensive analysis of both thermal and morphological dynamics of the melt pool. By leveraging the complementary strengths of infrared imaging (sensitivity to thermal changes) and visible light imaging (detailed spatial resolution), the proposed approach aims to provide a more meticulous and interference-resistant monitoring solution. The paper is organized as follows: Section 2 introduces the system setup and the data generation process; Section 3 analyzes the characteristics of visible and infrared images and the fusion network; Section 4 describes the training strategy and compares the network results. Finally, conclusions are drawn in Section 5.

## 2. System and Data Generation

### 2.1. Experiment System

The visible–infrared dual-modal defect detection system for WAAM, proposed in this paper, encompasses a front-end dual-modal data acquisition setup and a backend dual-input neural network model.

**Figure 1:**
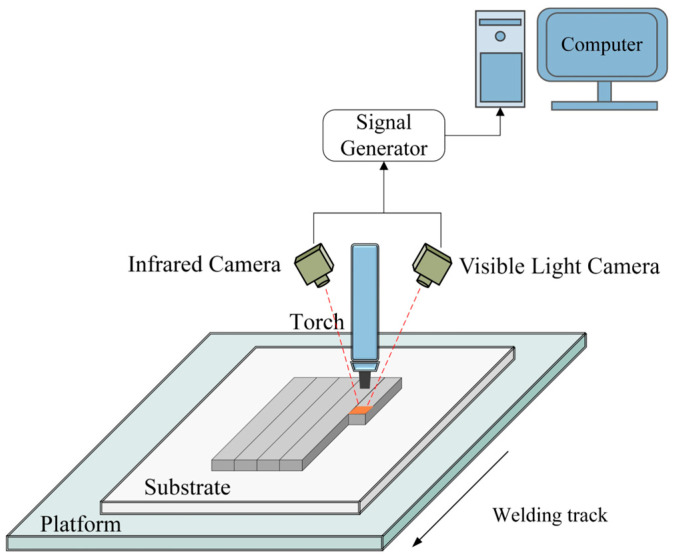
Visible–Infrared dual-modal acquisition system.

The front-end system, responsible for in-process data collection, is equipped with the following primary devices and their respective models: a six-axis robot (ABB IRB14000 M2004; ABB, Zürich, Switzerland), a CMT welding machine (Fronius CMT Advanced 4000R; Fronius International GmbH, Pettenbach, Austria), a monochrome visible light camera (Basler ace acA1920-155um; Basler AG, Ahrensburg, Germany),and an infrared camera (MAG3232006051; Shanghai Magnity Technologies Co., Ltd., Shanghai, China). Process monitoring, signal acquisition, and shielding-gas delivery were integrated within the welding control system. MAG3232006051 is a radiometric thermal camera (high-temperature version) with a resolution of 384 × 288 pixels, operating in the 7.5–14 μm spectral band at a native frame rate of 50 Hz, and a measurement range of 250–1600 °C. The camera supports radiometric calibration and emissivity correction; a constant emissivity setting was used during acquisition. An interchangeable lens was used, and the working distance to the melt pool was approximately 150 mm with a 45° view angle. Both the monochrome visible light and infrared cameras are mounted on the robotic arm angled at 45° to the torch, with a focus on the melt pool. Visible monochrome camera is fitted with 850 nm high pass filters to minimize arc light interference [39] (Figure 1).

During arc-based WAAM, the shielding gas flow and transient fume can intermittently scatter and attenuate optical signals, which reduces contrast in visible images and may introduce radiance fluctuations in passive infrared observations [40]. In this study, CMT was selected due to its comparatively low heat input and minimal spatter, which helps maintain a relatively stable optical field under normal operating conditions. In addition, the proposed dual-modal fusion is designed to help suppress modality-specific disturbances, improving robustness to such transient effects.

The experiments are conducted on 10 mm thick SS304 plates, with additional parameters outlined in Table 1. The process parameters in Table 1 were fixed intentionally, and defects were induced by increasing inter-track spacing so that the dataset isolates the lack of overlap mechanism instead of reflecting parameter driven variations. For the multimodal data fusion algorithm, temporal alignment of the data from different modalities is paramount. Thus, both cameras are configured in an external trigger mode, with a synchronized acquisition device sending signals to capture and transmit simultaneous dual-modal images of the melt pool back to the supervisory computer. The cameras operate at a fixed capture rate of 50 Hz.

### 2.2. Data Generation

**Figure 2:**
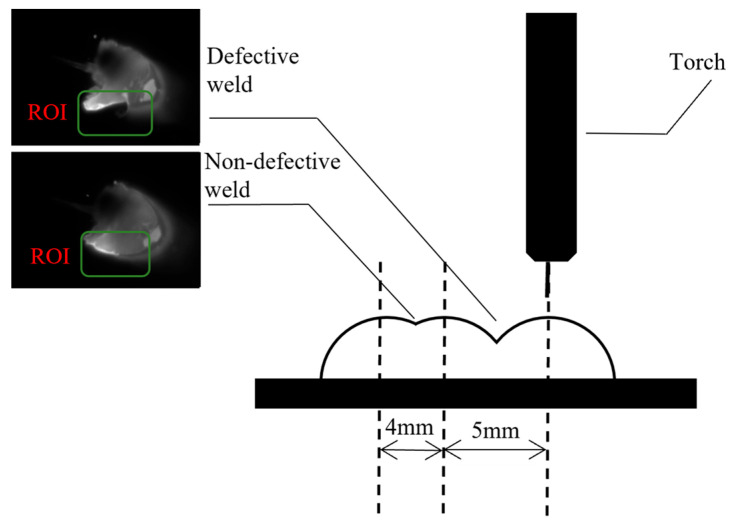
Schematic of overlap defect induction. Representative examples of non-defective and defective deposition are shown, where overlap defects are induced by increasing inter-track spacing.

In the WAAM process, improper settings of inter-track spacing or suboptimal welding parameters can lead to the occurrence of inter-run lack of overlap defects. To address these defects, a dataset was created where the initial two or three weld passes were conducted with a standard inter-track spacing (4 mm), followed by a wider spacing (5 mm) for the third or fourth pass to induce overlap defects. Throughout this procedure, current and voltage were consistently maintained (Figure 2).

**Figure 3:**
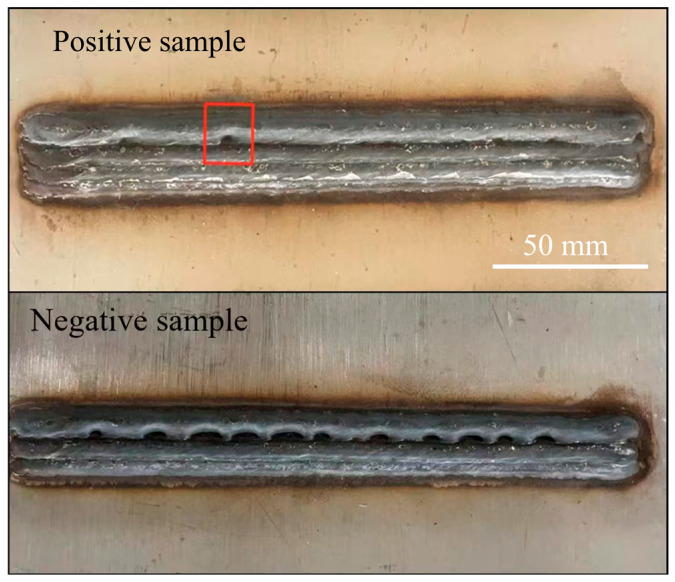
Representative positive and negative samples from additive experiments with scale bars. (**Top**): non-defective sample (positive class). (**Bottom**): defective sample (negative class) showing lack-of-overlap features. The deposited bead length is 200 mm. Occasional overlap-like features may also appear in normal welds (boxed in the top image); therefore, regions in the vicinity of these features were excluded during positive sample selection to avoid ambiguous labeling.

In the construction of the dataset for the dual-modal defect detection system, images captured during CMT process with poor overlap were labeled as negative samples. Conversely, those obtained from a welding process with proper overlap were designated as positive samples. It should be noted that normal WAAM processes may also yield instances of poor overlap, as illustrated in Figure 3. Samples adjacent to these defects were rigorously excluded to maintain the integrity of the dataset.

Acknowledging the variability inherent in the ignition and extinguishing phases of CMT, where the additive process is inherently unstable, dual-modal data acquired during these intervals were considered non-representative and thus discarded to avoid introducing extraneous noise into the dataset that could potentially disrupt model training.

The remaining data, consisting of 2100 frames of shuffled weld pass information, was randomly divided into a training set and a test set, accounting for approximately 84.18% and 15.82% of the data, respectively. This strategy ensures that the model is exposed to a comprehensive and representative sample of both defective and non-defective welding scenarios, enhancing its ability to generalize and accurately classify new data.

## 3. Dual-Modal Image Analysis and Modeling

### 3.1. Visible and Infrared Images

The design of Multimodal Mutual Fusion Network (MMFNet) is driven by the distinct characteristics of visible and infrared imaging modalities in the WAAM process. Advancements in visible light sensing technology allow for the acquisition of detailed morphological information of the melt pool. The morphology of the melt pool plays a vital role in real-time quality monitoring of WAAM, offering a direct visual indicator of the forming quality. Using morphological information from the melt pool, it is possible to monitor surface-level defects such as burrs, cracks, pores, and deviations.

**Figure 4:**
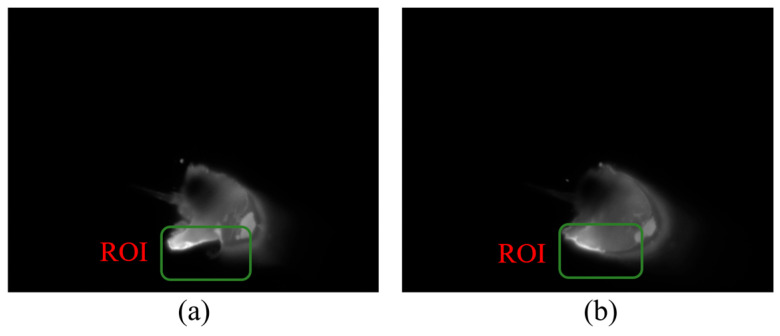
Visible light images of overlap defects. (**a**) Defective case and (**b**) non-defective case. The ROI is highlighted to emphasize the visual differences between the two cases.

Visible light melt pool images offer higher resolution, displaying the geometry of the melt pool including its shape, size, and potential surface disruptions indicative of defects such as poor overlap. These details are crucial for understanding the behavior of the molten material and the formation mechanisms of overlap defects. In cases of overlap defects, irregularities in the shape of the melt pool or evidence of discontinuity where the new layer has not properly fused with the previous layer may be observed. The Region of Interest (ROI) in Figure 4 shows the variation in the visible melt pool image when lap defects are present.

**Figure 5:**
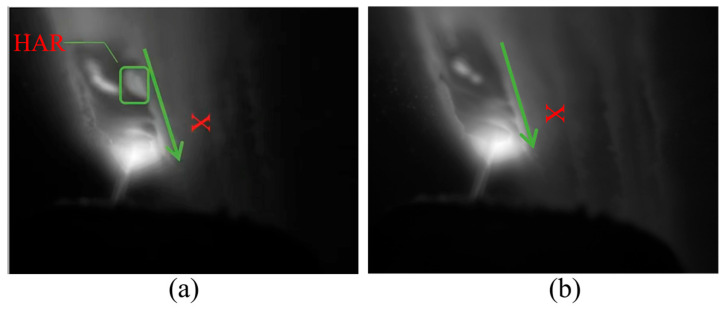
Infrared image of overlap defects. (**a**) Defective case and (**b**) non-defective case. The arrow indicates the deposition direction (x-axis) and the highlighted area shows the heat accumulation region used to emphasize the thermal difference between the two cases.

Particularly, poor inter-track overlap often manifests as anomalies within the thermal field. Incomplete bonding between adjacent tracks leads to reduced heat dissipation in the defect area, resulting in the formation of a heat accumulation region (HAR). Consequently, while normal weld seams display smooth temperature transitions, defect areas exhibit abnormal changes in the temperature curve, including areas of unusually high temperatures (as shown in Figure 5). A reliable quality monitoring system should therefore pay close attention to the thermal behaviors during the WAAM process, and infrared modal data are highly effective at capturing this thermal behavior information. Thus, beyond melt pool data, the incorporation of infrared thermal field information for in situ defect monitoring is crucial.

Infrared thermal field images reveal temperature distributions, providing insights into the thermal characteristics of the molten material. Infrared imaging can depict the temperatures of the melt pool and adjacent areas, with areas of insufficient overlap possibly displaying irregular thermal features. This could include abnormal hot spots where the material has not melted correctly, or cooler areas indicating incomplete melting or bonding.

**Figure 6:**
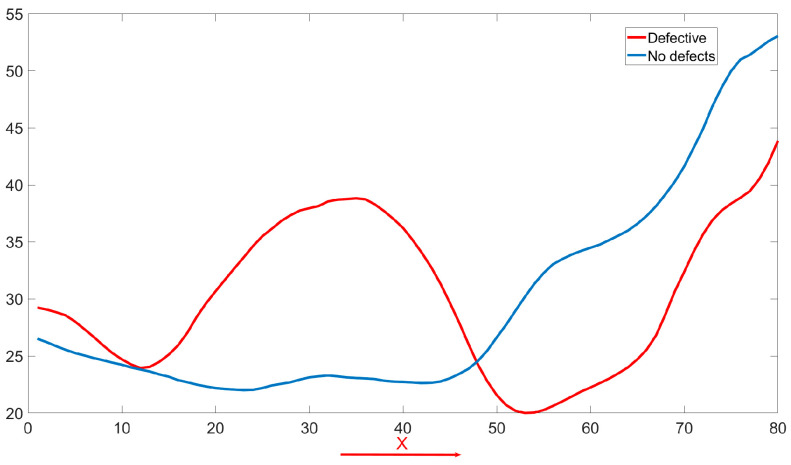
Temperature variation profiles of both weld beads along the x-direction (deposition direction).

In WAAM, the quality of interlayer bonding is crucial for the mechanical integrity of the final product. Infrared (IR) thermography serves as an effective diagnostic tool by providing real-time insights into thermal phenomena indicative of interlayer adhesion quality. Figure 5a displays the IR profile of a defective weld beads with a clearly delineated HAR, while Figure 5b shows defect-free weld beads, establishing a comparative benchmark. The temperature profile curve (Figure 6) highlights differences along the X-axis; defects related to inadequate overlap are marked by HAR, where incomplete bonding results in localized high temperatures. Conversely, a uniform heat distribution in defect-free weld beads indicates consistent interlayer fusion.

Therefore, a robust quality monitoring system should closely track these thermal behaviors. IR thermography not only enhances visible spectrum melt pool monitoring but is also essential for capturing critical heat flow and material consolidation details, vital for assessing quality. Integrating IR thermography into defect detection frameworks provides a comprehensive view of thermal dynamics, significantly improving the early detection of anomalies and thus enhancing the overall quality control.

**Figure 7:**
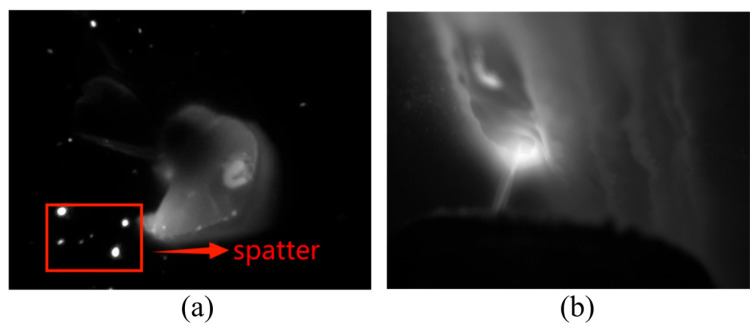
Representative visible and infrared melt pool images. (**a**) Visible light image of the melt pool, where spatter is highlighted as a typical optical disturbance. (**b**) Corresponding infrared image of the melt pool captured synchronously, providing complementary thermal information.

However, relying solely on any single modality presents inherent limitations: infrared imagery lacks detailed resolution, while visible light imagery is susceptible to interference from spatter and arc light, as demonstrated in Figure 7. Therefore, this study demonstrates the effectiveness of a multimodal approach, which leverages the complementary strengths of both infrared and visible light data, enhancing the capability to detect and analyze defects that may otherwise remain undetected with single-modal monitoring systems. Given the complementary nature of the two modalities, a fusion strategy is required that selectively enhances reliable features while suppressing noise. For instance, infrared thermal anomalies often correlate with visible morphological irregularities in overlap defects, but their spatial alignment may vary due to sensor placement and heat diffusion.

### 3.2. Fusion Strategies

The selection of an appropriate fusion strategy is crucial for successful multimodal integration. Currently, fusion strategies can be broadly categorized into early fusion and late fusion.

**Figure 8:**
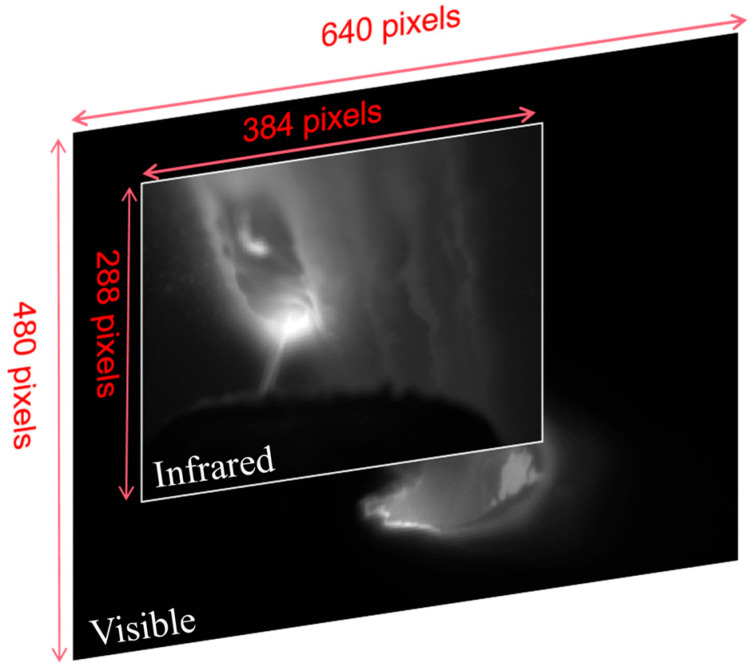
Difference in size between infrared temperature field data and visible light data. The visible image has a resolution of 640 × 480 pixels, while the infrared image has a resolution of 384 × 288 pixels. The inner box indicates the infrared field of view mapped onto the visible image for ROI alignment.

Early fusion involves merging different modal data at the very beginning of the processing pipeline, before inputting it into the neural network. This method is valued for its simplicity and straightforwardness, as it directly combines the raw data from all modalities into a single set that feeds into the learning algorithm. However, this approach imposes certain requirements on the data. In this case, the two modalities consist of image data acquired from visible light and infrared sensors. For effective early fusion, pixel-level registration of the two image types would be necessary. Due to sensor size limitations, there is a substantial resolution discrepancy between the infrared and visible light images, as shown in Figure 8. Achieving pixel-level registration would necessitate interpolation operations, which could introduce significant errors. Such resampling under cross-modal misalignment has been reported to cause structural artifacts such as ghosting and blur in infrared–visible fusion, making pixel-level early fusion sensitive to registration quality [41]. Meanwhile, the early fusion approach is prone to introduce inter-modal noise interference (e.g., arc light interference in the visible vs. thermal diffusion noise in the infrared). Related RGB–thermal perception studies have shown that naive early fusion can suffer from information interference between modalities and may not consistently outperform single-modality inputs [42]. In addition, the fixed fusion approach cannot dynamically adjust the modal contributions according to the mission requirements, resulting in amplified noise. Therefore, this study does not utilize early fusion.

Late fusion, on the other hand, refers to the integration of data at a later stage in the processing pipeline, often at the feature or decision level. Late fusion splicing or weighting at high-level features (e.g., in front of the classification layer) preserves modal independence but ignores fine-grained interaction information in the middle layer. For example, a mid-level feature in the visible may contain details of the melt pool profile, while an infrared feature may contain the thermal gradient distribution, and the two may not be effectively correlated at high levels due to loss of resolution.

**Figure 9:**
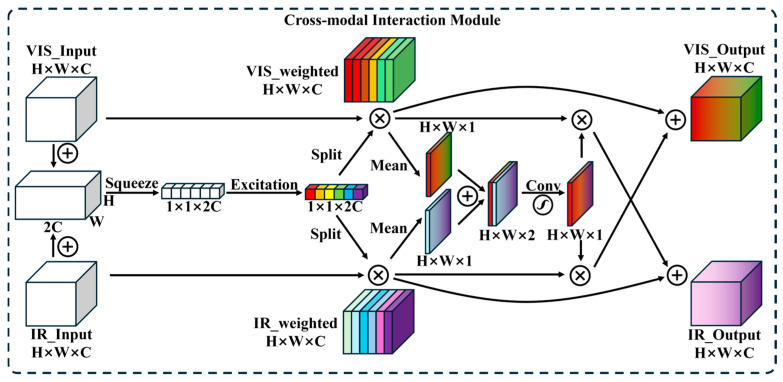
CMIM Structure.

To address these challenges, the Cross-Modal Interaction Module (CMIM) is proposed, which integrates channel and spatial attention mechanisms tailored for dual-modal data. The CMIM as an interlayer fusion strategy overcomes the noise sensitivity of early fusion and the fine-grained information loss problem of late fusion through layered dynamic fusion with dual-attention mechanism, striking a balance between accuracy, robustness and efficiency (Figure 9).

Joint Channel Attention: Unlike conventional Squeeze-and-Excitation (SE) modules that process modalities independently, visible and infrared features are concatenated before channel weight generation. This forces the model to learn inter-modal dependencies. For example, suppressing visible channels in arc light-saturated regions while amplifying infrared channels in corresponding thermal anomaly zones. The shared MLP reduces parameters by 25% compared to dual SE blocks while improving cross-modal synergy. The steps are as follows: let the input features from the visible and infrared branches be denoted as $F_{vis}$ and $F_{ir}$. Start by connecting the two features:

(1) $$F_{cat}=Concat(F_{vis},F_{ir})$$

The channel weights were generated after global average pooling (GAP) with Sigmoid function:

(2) $$W_{ch}=\sigma (\delta (GAP\left(F_{cat}\right)))$$

δ is the ReLU activation function, σ is the Sigmoid activation function. After weight splitting and feature recalibration the final channel attention features are obtained as Equation (4).

(3) $$W_{vis},W_{ir}=Split(W_{ch})$$

(4) $$\left\{\begin{array}{c} F_{vis}^{\prime }=F_{vis}⨀W_{vis}\\ F_{ir}^{\prime }=F_{ir}⨀W_{ir} \end{array}\right.$$

Here, ⨀ denotes channel-wise multiplication.

Cross-Modal Spatial Attention: Spatial weights are derived from concatenated channel-averaged maps of both modalities, allowing the model to learn where to emphasize complementary information. A 7 × 7 convolution followed by sigmoid activation generates the spatial attention map:

(5) $$\left\{\begin{array}{c} M_{vis}=\frac{1}{C}\sum_{c=1}^{C}F_{vis}^{\prime }[:,c,:,:]\\ M_{ir}=\frac{1}{C}\sum_{c=1}^{C}F_{ir}^{\prime }[:,c,:,:] \end{array}\right.$$

(6) $$W_{sp}=\sigma (Conv(Concat(M_{vis},M_{ir})))$$

The final output is the cross-modal feature fusion result:

(7) $$\left\{\begin{array}{c} F_{vis}^{out}=F_{vis}^{fused}=F_{vis}^{\prime }+W_{sp}⨀F_{ir}^{\prime }\\ F_{ir}^{out}=F_{ir}^{fused}=F_{ir}^{\prime }+W_{sp}⨀F_{vis}^{\prime } \end{array}\right.$$

This contrasts with independent spatial attention mechanisms, which may prioritize conflicting regions (e.g., focusing on spatter in visible images while ignoring thermal anomalies).

### 3.3. Network Framework

**Figure 10:**
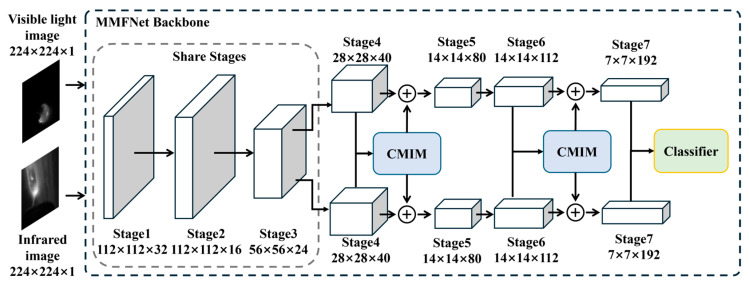
MMFNet framework.

Visible and infrared inputs are processed through two parallel EfficientNet-B0 backbones. Both modalities are resized to 256 × 256 before network input, and each modality is normalized independently to reduce sensor-dependent intensity scale differences. The EfficientNet-B0 backbones are trained from scratch in this study without ImageNet pretraining. The shallow layers (Stages 1–3) of both branches share weights to capture modality-agnostic low-level features such as edges and textures, which are critical for aligning the spatial characteristics of the melt pool across modalities. In contrast, the deeper layers (Stages 4–7) are trained independently to adapt to modality-specific patterns. For instance, the visible branch focuses on high-resolution morphological details (e.g., melt pool geometry and surface irregularities), while the infrared branch extracts thermal dynamics (e.g., HAR and temperature gradients). This design ensures efficient utilization of shared low-level features while preserving the unique discriminative capabilities of each modality (Figure 10).

To mitigate interference from arc light and spatter in visible images, CMIMs are embedded after Stages 4 and 6 to dynamically fuse cross-modal features. To balance detection accuracy and computational efficiency, only the final-stage features (Stage 7 outputs) from both modalities are concatenated for classification. While deeper layers sacrifice spatial resolution (7 × 7), they encode high-level semantic information critical for defect identification, such as global thermal patterns and consolidated morphological deviations. The fused features are passed through a lightweight classifier composed of global average pooling and two fully connected layers, achieving real-time inference while maintaining robustness against noise.

### 3.4. Loss Function

The model training relies on an optimization process that involving the search for the most favorable set of parameters within the confines of predetermined constraints. In the domain of deep learning, these constraints are encapsulated by what is termed a loss function. The loss function serves as a quantitative measure of the discrepancy between the predicted values and the true values, guiding the model’s predictions to align as closely as possible with reality.

In MMFNet, the total loss function consists of two key components: the Weighted Cross-Entropy Loss and the Cross-Modal Consistency Loss. These two loss functions are combined in a linearly weighted manner to synergistically optimize the model’s performance on both the classification task and the cross-modal feature alignment task.

The Weighted Cross-Entropy Loss for a binary classification task is defined as follows:

(8) $$L_{cls}=-\frac{1}{N}\sum_{i=1}^{N}\left[\alpha y_{i}\log\left(p_{i}\right)+(1-\alpha )(1-y_{i})\log\left(1-p_{i}\right)\right]$$

Here, $y_{i}$ represents the label for sample *i*, with 1 denoting the positive class and 0 the negative class. $p_{i}$ denotes the model’s predicted probability for sample *i* being of the positive class. α denotes the weight of the defective category (set to 0.7 in the experiment), which mitigates the category imbalance by boosting the loss contribution of defective samples. In the WAAM process, defective samples usually account for less than 10% of the samples, and the direct use of the standard cross entropy causes the model to be biased towards normal samples. Weighted cross-entropy enhances the focus on defects by adjusting the α value. It is evident that when the model’s predicted probability distribution aligns perfectly with the true one-hot vector, the cross-entropy loss function reaches its minimum value of 0. Conversely, greater discrepancies between the model’s predictions and the actual labels result in a larger loss function value, indicating a less accurate model.

Visible and infrared data may have skewed feature distributions due to sensor noise or physical property differences. By maximizing the cosine similarity of features in the middle layer, the model is forced to learn the semantic representation shared between modalities to improve the fusion robustness. Cross-Modal Consistency Loss acts on stage 4 and 6 with the goal of constraining the eigenspace alignment of the visible and infrared modes and reducing the effect of mode-specific noise. It is defined as follows:

(9) $$L_{cm}=\sum_{l\epsilon \{4,6\}}\left(1-cos(F_{vis}^{l},F_{ir}^{l})\right)$$

Here, l denotes the visible and infrared features of layer l (Stage 4 and Stage 6). cos (⋅) computed the cosine similarity, which measures the consistency of the direction of the feature vectors. The total loss function is the weighted sum of the above two losses:

(10) $$L_{total}=L_{cls}+\lambda L_{cm}$$

λ denotes the weighting factor for cross-modal consistency loss, which is determined by grid search, balancing classification performance with feature alignment strength. Each of the two loss functions has its own role: $L_{cls}$ dominates the optimization direction and ensures the core performance of the model on the defect detection task. $L_{cm}$ serves as a regularity term to prevent excessive deviation of inter-modal features and enhance the robustness of the model to noise.

## 4. Experimental Results and Analysis

### 4.1. Training Strategies

Pytorch was used to realize the above multimodal network architecture construction. NVIDIA RTX Titan (NVIDIA Corporation, Santa Clara, CA, USA) is used to train the multimodal model proposed in this paper and subsequent comparison tests. In the training phase, the mini-batch gradient descent algorithm is employed as the optimization algorithm, effectively bridging the gap between batch gradient descent and stochastic gradient descent. This method strikes a balance by incorporating the benefits of both—the computational efficiency of using batches and the stochastic approach’s noise reduction in gradient estimation.

Mini-batch gradient descent operates by randomly selecting a subset of training samples to compute gradients during each iteration. These gradients are then used to update the model parameters incrementally. The update rule for mini-batch gradient descent is articulated as follows:

(11) $$\theta_{t+1}=\theta_{t}-\alpha \frac{1}{m}\sum_{i=1}^{m}\left(h_{\theta }\left(x^{\left(i\right)}\right)-y^{\left(i\right)}\right)x^{\left(i\right)}$$

Here, *θ* represents the model parameters to be optimized, *α* signifies the learning rate, *m* denotes the size of the mini-batch, $h_{\theta }\left(x^{\left(i\right)}\right)$ is the predicted value by the model, $y^{\left(i\right)}$ is the true label of the sample, and $x^{\left(i\right)}$ is the feature vector of the sample.

The utilization of mini-batch gradient descent allows for a more controlled and potentially faster convergence on large datasets, making it a prudent choice for training deep learning models.

### 4.2. Ablation Study on Dual-Modal System

A systematic ablation study was conducted to quantify the contribution of the proposed MMFNet and to distinguish the benefit of dual-modal input from that of the fusion design. The collected paired images were randomly divided into training and validation sets with a 4:1 ratio, and all metrics were evaluated on the validation set only. Each configuration was trained and evaluated for five independent runs under the same setting to reflect run to run variability.

Table 2 compares three model configurations, including the visible-only model, the infrared-only model, and the complete dual-modal model. The infrared-only model achieves higher accuracy than the visible-only model, which is consistent with its ability to capture thermal anomalies related to incomplete inter-track bonding. The visible-only model shows larger variability, which is consistent with its higher sensitivity to transient optical disturbances such as arc glare and spatter. The dual-modal model provides the best overall performance, achieving the highest mean accuracy together with the smallest variability.

**Figure 11:**
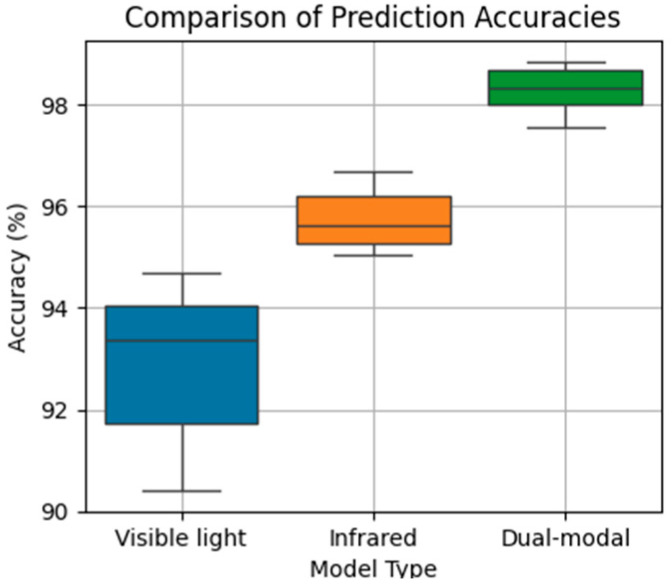
Box plot of three different models. The central line denotes the median, the box denotes the interquartile range, and the whiskers represent the range of the results. The spread reflects run-to-run variability under the same evaluation setting.

To make the run-to-run variability explicit, Figure 11 visualizes the distribution of validation accuracies over the five runs for the three configurations reported in Table 2. In this plot, the spread reflects variability across repeated training and evaluation under the same setting, rather than measurement uncertainty. The dual-modal model exhibits not only the highest median accuracy but also strong stability across all runs, with all results remaining above 98%.

Uncertainty of repeated evaluation:

To quantify the uncertainty associated with repeated evaluation, each configuration was trained and evaluated for n=5 independent runs under the same training and validation setting. Let $a_{i}$ denote the validation accuracy of run i. The mean accuracy is

$$\bar{a}=\frac{1}{n}\sum_{i=1}^{n}a_{n}$$

The sample standard deviation is

$$s=\sqrt{\frac{1}{n-1}\sum_{i=1}^{n}{\left(a_{i}-\bar{a}\right)}^{2}}$$

The standard uncertainty of the mean is

$$u\left(\bar{a}\right)=\frac{s}{\sqrt{n}}$$

and the expanded uncertainty at 95% confidence is

$$U=t_{0.975,n-1}u\left(\bar{a}\right)$$

where $t_{0.975,n-1}$ is the Student t factor for n−1 degrees of freedom. This uncertainty describes run-to-run variability due to stochastic training factors such as initialization and mini-batch ordering, and it does not represent sensor measurement uncertainty.

Using the results in Table 2, for the visible-only model $\bar{a}=92.85\%$ and s=1.64%, giving U≈2.04%. For the infrared-only model $\bar{a}=95.96\%$ and s=0.64%, giving U≈0.79%. For the dual-modal model $\bar{a}=98.34\%$ and s=0.54%, giving U≈0.67%. These results indicate that the dual-modal model not only achieves the highest mean accuracy but also exhibits lower run-to-run variability than the unimodal baselines under the same evaluation setting.

To isolate the effect of the proposed CMIM, Table 3 reports a dual-modal baseline without CMIM and the complete dual-modal model. The baseline without CMIM improves accuracy relative to unimodal models, confirming that combining infrared and visible information enhances robustness beyond any single source. Adding CMIM further increases accuracy from 96.05% to 98.34% demonstrating that the performance gain is not solely due to the availability of dual-modal input, but is also attributable to the proposed interactive fusion mechanism.

The per-class metric further confirms the benefit of CMIM for defect-risk reduction. As shown in Table 3, incorporating CMIM improves recall from 0.90 to 0.95, indicating fewer missed-defect cases under the same evaluation setting. This improvement complements the overall accuracy gain and supports the effectiveness of the proposed interactive fusion mechanism for robust overlap defect detection.

In summary, the ablation results verify that the performance superiority of the proposed system is intrinsically linked to the CMIM-based fusion architecture. The CMIM is the key component that unlocks the synergistic potential of dual-modal data for robust overlap defect detection.

### 4.3. Comparative Analysis

Having established the efficacy of the fusion strategy, the proposed MMFNet was benchmarked against state-of-the-art network architectures to evaluate its overall performance. The competitors included widely adopted models in the field: ResNet-50 and Vision Transformer (ViT-Base). To ensure a fair comparison, all models were adapted to accept dual-modal input via early fusion (channel-wise concatenation of visible and infrared images at the input layer), which is the most common baseline approach for multimodal learning.

**Figure 12:**
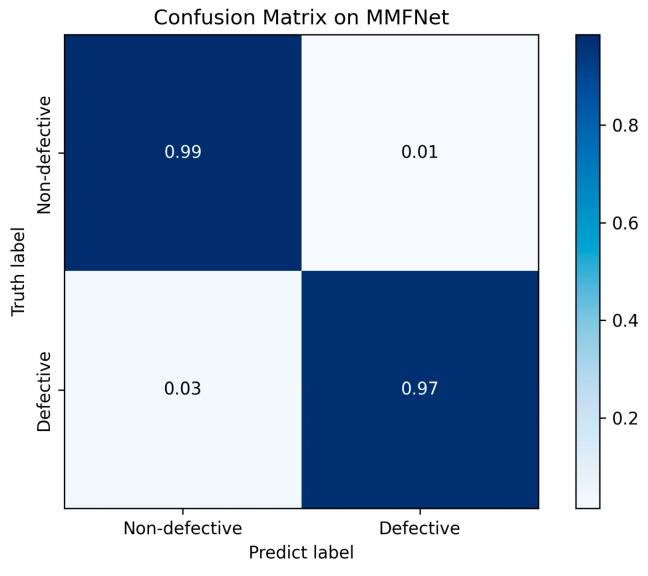
Confusion matrices of Dual-Modal.

Figure 12 presents the confusion matrices corresponding to the binary classification task under different input configurations. The diagonal entries of each matrix indicate correctly classified instances, while the off-diagonal elements represent misclassifications. A higher concentration along the diagonal signifies improved classification accuracy, thereby providing an intuitive visualization of the comparative performance across unimodal and multimodal models.

(12) $$F_{1}=2\times \frac{Precision\times Recall}{Precision+Recall}$$

(13) $$Macro\_F_{1}=\frac{1}{n}\sum_{i=1}^{n}F_{1}(i)$$

The F1 score, computed for each class individually, serves as a balanced measure of classification performance. It is defined as the harmonic mean of precision and recall, effectively capturing the trade-off between false positives and false negatives. A higher F1 score indicates that the model achieves both high precision and high recall, making it particularly suitable for evaluating performance in imbalanced classification scenarios.

The comparative results are presented in Table 4. The proposed MMFNet model obtained the highest scores across all evaluated metrics. It achieved an accuracy of 98.34%, compared to 95.36% for ResNet-50 and 91.39% for ViT. More notably, the Recall rate for MMFNet was 95.00%, exceeding that of ResNet-50 and ViT. Given that Recall measures the model’s ability to identify all actual defect instances, a higher value is critical for minimizing missed detections in quality monitoring applications.

The Macro-F1 score, which provides a balanced summary of precision and recall, was 99.11% for MMFNet. This represents an increase of 4.55% over ResNet-50 (94.56%) and 9.40% over ViT (89.71%). The superior Macro-F1 score indicates that the proposed model maintains a more robust balance between correctly identifying defects and minimizing false alarms, even in the presence of class imbalance.

The results in Table 4 indicate that while general-purpose architectures like ResNet and ViT provide strong baseline performance, a specialized design may be better suited for the specific requirements of multimodal defect detection. The early fusion strategy employed for the baseline models may be insufficient for modeling the complex, non-linear interactions between visible and infrared modalities. In contrast, the MMFNet architecture, which incorporates dedicated backbones and cross-modal interaction modules, appears to more effectively leverage the complementary information present in the two data streams.

### 4.4. Discussion

**Figure 13:**
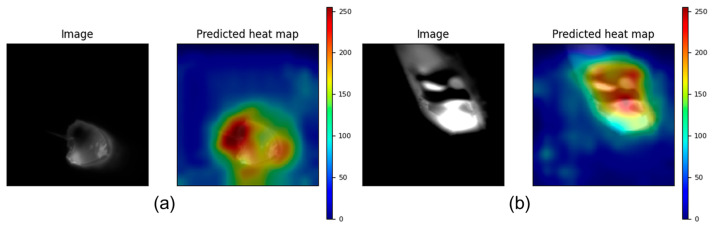
Class Activation Mapping results: (**a**) MMFNet attention on visible image, (**b**) MMFNet attention on infrared image.

The experimental results demonstrate that the dual-modal system achieves a significant improvement in overlap defect detection accuracy compared to single-modality models. Infrared imaging’s superiority in capturing thermal anomalies aligns with its ability to directly characterize temperature gradients caused by incomplete inter-track bonding. This advantage is visually corroborated by the Class Activation Mapping (CAM) analysis, which reveals that the infrared modality generates highly focused activation patterns concentrated specifically within the heat-affected zone. The infrared CAM exhibits a notable insensitivity to arc light interference, allowing it to maintain precise localization on thermally anomalous regions that correspond to areas of incomplete bonding and abnormal heat retention (Figure 13).

Complementing the thermal analysis, visible light imaging provides distinct morphological information essential for characterizing geometric aspects of defects. The CAM visualization for the visible modality demonstrates a fundamentally different attention pattern, with activation distributed across the overall bead contour and particularly concentrated at the trailing edge of the melt pool. This focus on geometrical transitions and boundary variations captures critical morphological evidence of overlap defects, though the modality remains susceptible to arc light artifacts that can disperse attention patterns.

By synergizing these complementary modalities, the proposed system effectively leverages the precise thermal localization of infrared imaging and the detailed morphological sensitivity of visible light. The CAM analysis provides compelling visual evidence of this complementary integration: while infrared attention pinpoints the thermal signature within the HAR, visible light attention captures the geometrical manifestations at bead boundaries and melt pool transitions. This dual-perspective approach enables comprehensive defect characterization that neither modality could achieve independently, explaining the model’s superior performance and low variability in accuracy across trials.

In terms of computational efficiency, the current implementation provides a practical baseline for online monitoring. On the present hardware, the average inference time of the dual-modal model is 41.7 ms per paired frame. This indicates that the method can support online analysis at a reduced acquisition rate, while end-to-end latency benchmarking and validation under representative industrial conditions remain for future work.

### 4.5. Limitations and Scope

This study focuses on multi-pass WAAM builds and the overlap defect induced by inter-track spacing, and the current validation is limited to this defect scenario. Although arc-based WAAM is generally subject to optical disturbances related to shielding gas flow and transient fume, the dataset in this work was collected under stable and normal CMT operating conditions and does not intentionally cover cases with dense fume or severe optical occlusion. Therefore, the robustness of the proposed system under harsher industrial environments, such as poor ventilation, heavy fume, strong ambient illumination, or mechanical vibration, remains to be validated.

In addition, the experiments were conducted using a single baseline process parameter set in order to induce the target defect mechanism, rather than to map the full parameter space. The proposed model is intended to learn thermal and morphological anomalies associated with insufficient overlap, and generalization across broader process windows and different materials has not yet been systematically verified. Future work will expand validation to broader operating conditions, additional defect types, and more diverse build settings. Future research will also investigate cost-effective alternatives to infrared cameras, such as enhanced visible light sensors with broader temperature response ranges (see Appendix A for preliminary analysis). If real-time performance claims are required, end-to-end latency and inference throughput should be measured and reported under representative deployment hardware.

## 5. Conclusions

This study has established a visible–infrared dual-modal monitoring system for detecting overlap defects in wire arc additive manufacturing. The principal conclusions are as follows:

(1) A dual-modal fusion network, MMFNet, was designed around a Cross-Modal Interaction Module that performs dynamic feature-level fusion. Ablation studies verified that this module is critical for performance, as it effectively suppresses modality-specific noise and enhances complementary information.
(2) The proposed MMFNet architecture, incorporating a dedicated CMIM, achieves a defect detection accuracy of 98.34%. This represents a significant improvement over comparable unimodal models, which attained accuracies of 95.76% (infrared) and 92.85% (visible light). The network’s design facilitates effective feature-level fusion of complementary information.
(3) The model demonstrated high robustness, with low performance variance across trials. Visualization via Class Activation Mapping provided interpretable evidence that the model’s decisions are based on physically relevant features, including thermal anomalies in the infrared modality and morphological irregularities in the visible modality.

In summary, this work validates the significant advantage of intelligent multimodal fusion for quality assurance in additive manufacturing. The proposed framework offers a reliable and interpretable solution for detecting overlap defects.

Future work will focus on validating robustness under representative industrial conditions, including stronger optical disturbances and longer production runs. In addition, expanding the dataset to cover broader process windows, different materials, and additional defect types will be important to assess generalization. Finally, system-level benchmarking and optimization will be conducted to quantify end-to-end latency and to support online monitoring in deployment-oriented settings.

## Figures and Tables

**Table 1:** Experimental parameter setting.

Welding Parameters	
Base material	SS304
Wire diameter (mm)	1.2
Wire feed speed (m/min)	5.6
Welding speed (mm/s)	7
Welding length (mm)	200
Welding Current (A)	144
Lifting height of torch (mm)	3
Distance between bead (mm) (1st–2nd or 3rd bead)	4
Distance between bead (mm) (3rd or 4th bead)	5
Shielding gas	Ar (98.5%) + O_2_ (1.5%)
Gas flow (L/min)	25
Camera exposure time (ms)	0.15
Camera acquisition frequency (Hz)	50
Visible camera to torch angle	45°
Distance between visible camera and torch (mm)	150

**Table 2:** Accuracy of three model configurations over five independent runs.

Trial	Visual	Infrared	Dual-Modal
1st	94.70%	96.67%	98.68%
2nd	94.04%	96.21%	98.01%
3rd	93.38%	95.63%	98.84%
4th	90.42%	95.03%	97.56%
5th	91.72%	96.27%	98.61%
Avg acc	92.85%	95.96%	98.34%
Std	1.64%	0.64%	0.54%

**Table 3:** Ablation results on the CMIM using dual modal input.

Metric	Without CMIM	Dual-Modal
Accuracy	96.05%	98.34%
Defect Recall	90%	95%

**Table 4:** Comparison experiment results.

Network	Accuracy (%)	Recall (%)	Macro-F1 (%)
ResNet	95.36	86	94.56
ViT	91.39	77	89.71
MMFNet	98.34	95	99.11

**Figure A1:**
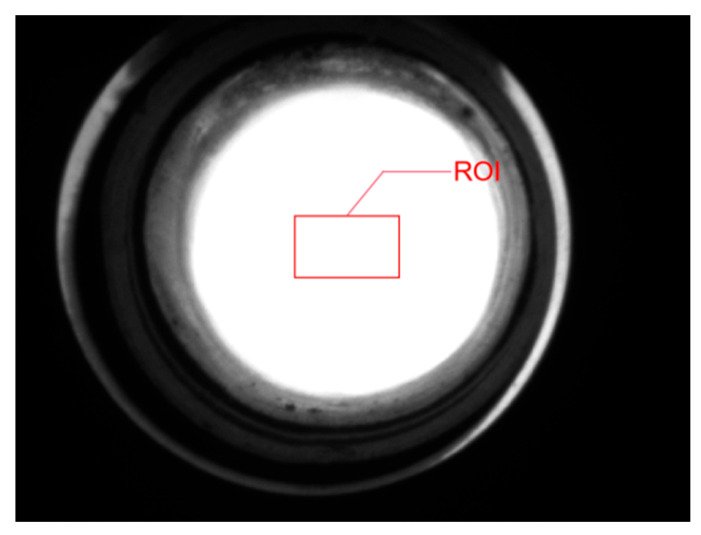
ROI selection in blackbody furnace images for grayscale-temperature correlation.

**Figure A2:**
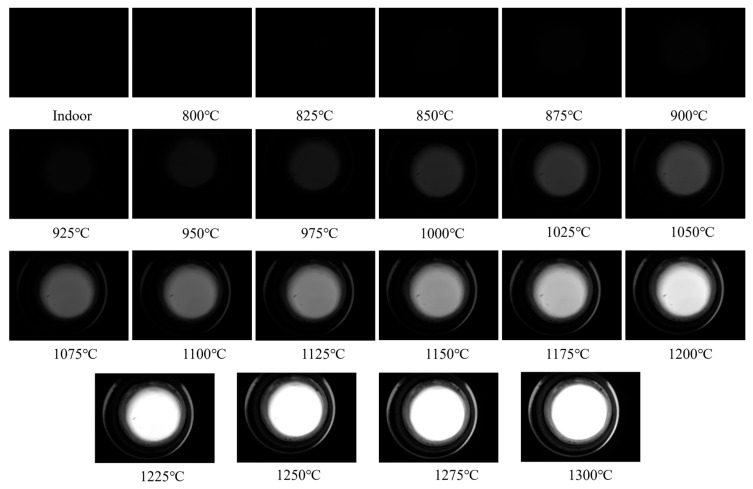
Visible light camera images of the blackbody furnace at 800–1300 °C. Saturation occurs beyond 1225 °C (bright regions).

**Figure A3:**
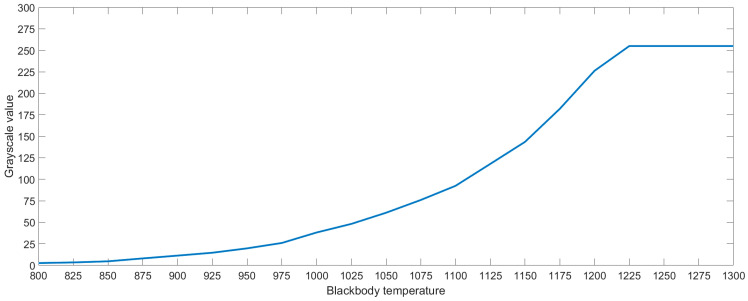
Grayscale temperature response curve of the visible light camera, showing a limited measurement range (825–1225 °C).

## Data Availability

The raw data supporting the conclusions of this article will be made available by the authors on request.

## Appendix A. Visible Light Camera Temperature Calibration

### Appendix A.1. Objective

This appendix evaluates the temperature measurement capability of the monochrome visible light camera used in the dual-modal system. The goal was to assess its potential as a low-cost alternative to infrared thermography for thermal field characterization.

### Appendix A.2. Methods

A blackbody furnace (Model BBX-1200) was employed to calibrate the camera under controlled conditions. The camera’s exposure time (0.15 ms) and aperture settings matched those used in the WAAM experiments (Table 1). Images were captured at temperatures ranging from 800 °C to 1300 °C. A fixed region of interest (ROI) was selected to calculate the average grayscale value, which was correlated with temperature (Figure A1).

### Appendix A.3. Results

The visible light camera exhibited no response below 825 °C and reached saturation beyond 1225 °C (Figure A2). Within this narrow range (825–1225 °C), grayscale values showed a near-linear relationship with temperature (Figure A3). These limitations indicate that visible light imaging alone cannot fully replace infrared thermography for comprehensive thermal monitoring in WAAM.

## References

[1] Chu H.H., Wang Z.Y. (2016). A vision-based system for post-welding quality measurement and defect detection. Int. J. Adv. Manuf. Technol..

[2] Biswas P., Sarker B.R. (2008). Optimal batch quantity models for a lean production system with in-cycle rework and scrap. Int. J. Prod. Res..

[3] Bahr N.J. (2014). System Safety Engineering and Risk Assessment: A Practical Approach.

[4] Zhang Y., Shen S., Li H., Hu Y. (2022). Review of in situ and real-time monitoring of metal additive manufacturing based on image processing. Int. J. Adv. Manuf. Technol..

[5] Sumesh A., Rameshkumar K., Raja A., Mohandas K., Santhakumari A., Shyambabu R. (2017). Establishing Correlation Between Current and Voltage Signatures of the Arc and Weld Defects in GMAW Process. Arab. J. Sci. Eng..

[6] Thekkuden D.T., Mourad A.H.I. (2019). Investigation of feed-forward back propagation ANN using voltage signals for the early prediction of the welding defect. SN Appl. Sci..

[7] Santoro L., Sesana R., Molica Nardo R., Curá F. (2023). Infrared in-line monitoring of flaws in steel welded joints: A preliminary approach with SMAW and GMAW processes. Int. J. Adv. Manuf. Technol..

[8] Yu R., Huang Y., Peng Y., Wang K. (2023). Monitoring of butt weld penetration based on infrared sensing and improved histograms of oriented gradients. J. Mater. Res. Technol..

[9] Wang Y., Lee W., Jang S., Truong V., Jeong Y., Won C., Lee J., Yoon J. (2024). Prediction of internal welding penetration based on IR thermal image supported by machine vision and ANN-model during automatic robot welding process. J. Adv. Join. Process..

[10] Zhao Z., Lv N., Xiao R., Liu Q., Chen S. (2023). Recognition of penetration states based on arc sound of interest using VGG-SE network during pulsed GTAW process. J. Manuf. Process..

[11] Chen L., Yao X., Tan C., He W., Su J., Weng F., Chew Y., Ng N., Moon S. (2023). In-situ crack and keyhole pore detection in laser directed energy deposition through acoustic signal and deep learning. Addit. Manuf..

[12] Yusof M.F.M., Quazi M.M., Aleem S.A.A., Ishak M., Ghazali M. (2023). Identification of weld defect through the application of denoising method to the sound signal acquired during pulse mode laser welding. Weld. World.

[13] Shao J., Yu G., He X., Li S., Li Z., Wang X. (2021). Process maps and optimal processing windows based on three-dimensional morphological characteristics in laser directed energy deposition of Ni-based alloy. Opt. Laser Technol..

[14] Lane B., Zhirnov I., Mekhontsev S., Grantham S., Ricker R., Rauniyar S., Chou K. (2020). Transient Laser Energy Absorption, Co-axial Melt Pool Monitoring, and Relationship to Melt Pool Morphology. Addit. Manuf..

[15] Kanko J.A., Sibley A.P., Fraser J.M. (2016). In situ morphology-based defect detection of selective laser melting through inline coherent imaging. J. Mater. Process. Technol..

[16] Wang H., Li B., Zhang S., Xuan F. (2024). Traditional machine learning and deep learning for predicting melt-pool cross-sectional morphology of laser powder bed fusion additive manufacturing with thermographic monitoring. J. Intell. Manuf..

[17] Myers A.J., Quirarte G., Ogoke F., Lane B.M., Uddin S.Z., Farimani A.B., Beuth J.L., Malen J.A. (2023). High-resolution melt pool thermal imaging for metals additive manufacturing using the two-color method with a color camera. Addit. Manuf..

[18] García-Moreno A.-I., Alvarado-Orozco J.-M., Ibarra-Medina J., Martínez-Franco E. (2021). In-process monitoring of the melt-pool motion during continuous-wave laser metal deposition. J. Manuf. Process..

[19] Yu R., Kershaw J., Wang P., Zhang Y. (2022). How to Accurately Monitor the Weld Penetration From Dynamic Weld Pool Serial Images Using CNN-LSTM Deep Learning Model?. IEEE Robot. Autom. Lett..

[20] Yu R., Cao Y., Chen H., Ye Q., Zhang Y. (2023). Deep learning based real-time and in-situ monitoring of weld penetration: Where we are and what are needed revolutionary solutions?. J. Manuf. Process..

[21] Xu P., Zhu X., Clifton D.A. (2023). Multimodal Learning With Transformers: A Survey. IEEE Trans. Pattern Anal. Mach. Intell..

[22] Ouhaichi H., Spikol D., Vogel B. (2023). Research trends in multimodal learning analytics: A systematic mapping study. Comput. Educ. Artif. Intell..

[23] Alibeigi M., Ljungbergh W., Tonderski A., Hess G., Lilja A., Lindstr¨om C., Motorniuk D., Fu J., Widahl J., Petersson C. Zenseact Open Dataset: A large-scale and diverse multimodal dataset for autonomous driving. Proceedings of the IEEE International Conference on Computer Vision.

[24] Yang J., Liang N., Pitts B.J., Prakah-Asante K.O., Curry R., Blommer M. (2023). Multimodal Sensing and Computational Intelligence for Situation Awareness Classification in Autonomous Driving. IEEE Trans. Hum.-Mach. Syst..

[25] Cao L., Zhang H., Peng C., Hansberger J.T. (2023). Real-time multimodal interaction in virtual reality—A case study with a large virtual interface. Multimed. Tools Appl..

[26] Zhang Z., Chen S. (2017). Real-time seam penetration identification in arc welding based on fusion of sound, voltage and spectrum signals. J. Intell. Manuf..

[27] Liu H., Gobert C., Ferguson K., Abranovic B., Chen H., Beuth J.L., Rollett A.D., Kara L.B. (2024). Inference of highly time-resolved melt pool visual characteristics and spatially-dependent lack-of-fusion defects in laser powder bed fusion using acoustic and thermal emission data. Addit. Manuf..

[28] Chen L., Yao X., Feng W., Chew Y., Moon S.K. Multimodal Sensor Fusion for Real-Time Location-Dependent Defect Detection in Laser-Directed Energy Deposition. Proceedings of the ASME Design Engineering Technical Conference.

[29] Wu D., Huang Y., Zhang P., Yu Z., Chen H., Chen S. (2020). Visual-Acoustic Penetration Recognition in Variable Polarity Plasma Arc Welding Process Using Hybrid Deep Learning Approach. IEEE Access.

[30] Gao P., Su X., Wu Z., Lu J., Han J., Bai L., Zhao Z. (2024). Online penetration prediction based on multimodal continuous signals fusion of CMT for full penetration. J. Manuf. Process..

[31] Quackatz L., Gornushkin I., Griesche A., Kannengiesser T., Treutler K., Wesling V. (2024). In situ chemical analysis of duplex stainless steel weld by laser induced breakdown spectroscopy. Spectrochim. Acta-Part B At. Spectrosc..

[32] Gött G., Gericke A., Henkel K.M., Uhrlandt D. (2016). Optical and spectroscopic study of a submerged arc welding cavern. Weld. J..

[33] Chen X., Sun B., Zhang C., Lou X., Zhao Z., Han J. (2020). Wire composition and shielding gas flow monitoring based on image and spectrum multimodal network. Meas. J. Int. Meas. Confed..

[34] Kang S., Kang M., Hoon Jang Y., Kim C. (2024). Spectrometer as a quantitative sensor for predicting the weld depth in laser welding. Opt. Laser Technol..

[35] Zhang Y., You D., Gao X., Zhang N., Gao P.P. (2019). Welding defects detection based on deep learning with multiple optical sensors during disk laser welding of thick plates. J. Manuf. Syst..

[36] Vadidar M., Kariminezhad A., Mayr C., Kloeker L., Eckstein L. Robust Environment Perception for Automated Driving: A Unified Learning Pipeline for Visual-Infrared Object Detection. Proceedings of the IEEE Intelligent Vehicles Symposium.

[37] Xu H., Huang H. (2023). In situ monitoring in laser melt injection based on fusion of infrared thermal and high-speed camera images. J. Manuf. Process..

[38] Jiang R., Xiao R., Chen S. (2021). Prediction of penetration based on infrared thermal and visual images during pulsed GTAW process. J. Manuf. Process..

[39] Lenef A.L., Gardner C.S. (1985). Optical emissions from weld arcs and their effects on the performance of welding robot vision systems. Appl. Opt..

[40] Cai L., Zhao H. (2025). A Multimodal Fusion Method for Weld Seam Extraction Under Arc Light and Fume Interference. J. Manuf. Mater. Process..

[41] Wang D., Liu J., Fan X., Liu R. Unsupervised Misaligned Infrared and Visible Image Fusion via Cross-Modality Image Generation and Registration. Proceedings of the IJCAI International Joint Conference on Artificial Intelligence.

[42] Zhang X., Cao S.Y., Wang F., Zhang R., Wu Z., Zhang X., Bai K., Shen H.-L. (2025). Rethinking Early-Fusion Strategies for Improved Multispectral Object Detection. IEEE Trans. Intell. Veh..

